# Changing epidemiology of catheter-related bloodstream infections in neutropenic oncohematological patients

**DOI:** 10.1371/journal.pone.0251010

**Published:** 2021-04-30

**Authors:** Dajana Lendak, Pedro Puerta-Alcalde, Estela Moreno-García, Mariana Chumbita, Nicole García-Pouton, Celia Cardozo, Laura Morata, Maria Suárez-Lledó, Marta Hernández-Meneses, Lucio Ghiglione, Francesc Marco, Jose Antonio Martinez, Josep Mensa, Ivana Urošević, Alex Soriano, Carolina Garcia-Vidal

**Affiliations:** 1 Faculty of Medicine, University of Novi Sad, Novi Sad, Serbia; 2 Clinical Centre of Vojvodina, Clinic for Infectious Diseases, Novi Sad, Serbia; 3 Infectious Diseases Department, Hospital Clínic-IDIBAPS, Barcelona, Spain; 4 Hematology Department, Hospital Clínic, Barcelona, Spain; 5 Oncology Department, Hospital Clínic, Barcelona, Spain; 6 Microbiology Department, Centre Diagnòstic Biomèdic, Hospital Clínic, Barcelona, Spain; 7 ISGlobal, Hospital Clínic—Universitat de Barcelona, Barcelona, Spain; 8 University of Barcelona, Barcelona, Spain; 9 Clinical centre of Vojvodina, Clinic for Haematology, Novi Sad, Serbia; University of Nicolaus Copernicus in Torun, POLAND

## Abstract

**Background:**

We aimed to describe the epidemiology of catheter-related bloodstream infections (CRBSIs) in onco-hematological neutropenic patients during a 25-year study period, to evaluate the risk factors for Gram-negative bacilli (GNB) CRBSI, as well as rates of inappropriate empirical antibiotic treatments (IEAT) and mortality.

**Materials/Methods:**

All consecutive episodes of CRBSIs were prospectively collected (1994–2018). Changing epidemiology was evaluated comparing five-year time spans. A multivariate regression model was built to evaluate risk factors for GNB CRBSIs.

**Results:**

482 monomicrobial CRBSIs were documented. The proportion of CRBSIs among all BSIs decreased over time from 41.2% to 15.8% (p<0.001). CRBSIs epidemiology has been changing: the rate of GNB increased over time (from 11.9% to 29.4%; p<0.001), as well as the absolute number and rate of multidrug-resistant (MDR) GNB (from 9.5% to 40.0%; p = 0.039). *P*. *aeruginosa* increased and comprised up to 40% of all GNB. Independent factors related with GNB-CRBSIs were: longer duration of in-situ catheter (OR 1.007; 95%CI 1.004–1.011), older age (OR 1.016; 95%CI 1.001–1.033), prior antibiotic treatment with penicillins (OR 2.716; 95%CI 1.306–5.403), and current antibiotic treatment with glycopeptides (OR 1.931; 95%CI 1.001–3.306). IEATs were administered to 30.7% of patients, with the highest percentage among MDR *P*. *aeruginosa* (76.9%) and *S*. *maltophillia* (92.9%). Mortality rate was greater among GNB than GPC-CRBSI (14.4% vs 5.4%; p = 0.002), with mortality increasing over time (from 4.5% to 11.2%; p = 0.003).

**Conclusion:**

A significant shift towards GNB-CRBSIs was observed. Secondarily, and coinciding with an increasing number of GNB-MDR infections, mortality increased over time.

## Introduction

Severe immunosuppression, chemotherapy regimens, bone marrow suppression, and transplantation, as well as indwelling intravascular catheters, are recognized as etiological factors for increased risk of infection in patients with hematological and oncological malignancies [1,2]. Also, a potential relation of insertion site and catheter-related complications has been reported [3,4]. Catheter-related bloodstream infections (CRBSI) are one of the most important infection in those patients, causing up to almost half of all bloodstream infections (BSI), and incidence rates ranging from 10 to 18 per 1000 central venous catheter days [5,6]. Besides the clinical impact, CRBSI are associated with prolonged hospitalizations, significant morbidity and increased health-care costs [7]. Finally, mortality rates between 12 and 40% are reported [8].

We hypothesize that the epidemiology of catheter infections has changed due to the constant dynamic modifications in catheter management and hospital ecology. Current information regarding the epidemiology of CRBSI in onco-hematological patients is scarce. The leading guidelines for management of CRBSI suggest introducing antibiotics for Gram-negative (GN) coverage if the patient is neutropenic [9,10], but some concerns are related to this practice. First, the changes in CRBSI epidemiology, as it concerns the specific population of onco-hematological patients have not been well described. Second, risk factors for Gram-negative bacilli (GNB) CRBSIs have not been identified. Finally, it remains unknown how the emergence of multidrug-resistant GNB infections worldwide impact catheter-related infections in this particular population. Consequently, the optimal empirical antibiotic strategy to cover a potential GN infection has yet to be well established.

This study aimed to describe the incidence and epidemiology of CRBSI in neutropenic onco-hematological patients during a 25-year study period, and evaluate the risk factors for GNB-CRBSIs, as well as rates of inappropriate empirical antibiotic treatment (IEAT) and mortality.

## Materials and methods

### Setting and data collection

This study was performed at Hospital Clinic of Barcelona, throughout a 25-yearperiod (1994–2018). Hospital Clinic of Barcelona is a 700-bed university hospital that attends to approximately 500,000 inhabitants within an urban setting.

### Study population and design

This study included all consecutive episodes of BSI in neutropenic onco-hematological patients. In order to compare characteristics of the BSI episodes, the 25-year period was divided into five different 5-year time-spans. Data were prospectively collected and retrospectively analyzed.

Decisions about the need for catheter placement and the use of specific access were made at the discretion of patients’ doctors. Catheters used were non-antimicrobial-coated and were inserted by physicians per standard sterile barrier precautions. Catheter site and adequate functioning were routinely inspected by nurses in each shift and replaced in the case of apparent malfunctioning or signs of complications.

The study was approved by the Ethical Committee Board of Hospital Clinic of Barcelona. Informed consent was waived because of the retrospective design of the study and all data being fully anonymized.

### Definitions

Neutropenia was defined as an absolute neutrophil count of <500 cells/mm^3^. Catheter-related BSI was defined according to the guidelines from Infectious Disease Society of America (IDSA) [9]: Bacteremia or fungemia in a patient with an intravascular device and ≥1 positive blood culture result obtained from the peripheral vein, clinical manifestations of infection (e.g., fever, chills, and/or hypotension), and no apparent source of BSI (with the exception of the catheter). One of the following should be present: a positive result of semi-quantitative (>15 colony-forming unit [CFU] per catheter segment) or quantitative (>10^2^ CFU per catheter segment) catheter culture, whereby the same organism (species) is isolated from the catheter segment and the peripheral blood culture; simultaneous quantitative cultures of blood with a ratio of 13:1 CFU/mL of blood (catheter vs. peripheral blood); differential time to positivity (growth in a culture of blood obtained through a catheter hub is detected by an automated blood culture system at least 2h prior than that of a culture of simultaneously drawn peripheral blood of equal volume).

Prognosis of underlying disease was defined according to McCabe and Jackson modified criteria: non-fatal (if life expectancy is longer than 5 years), ultimately fatal (if death is expected within a period of more than 3 months but less than 5 years) and rapidly fatal (death expected within 3 months) [11]. Shock was defined as systolic pressure *<*90mmHg that was unresponsive to fluid treatment or required vasoactive drug therapy. Prior antibiotic treatment was defined as the use of any antimicrobial agent for more than 3 days in the month prior to the BSI onset. According to the hospital protocols, patients with the expected duration of neutropenia longer than 10 days received prophylaxis by two different strategies during the evaluated period. From 1994 to 1995, all patients received an oral combination of amikacin, vancomycin, and colimicin for intestinal decontamination. In 1995, these agents were replaced by a fluoroquinolone (Ciprofloxacin or Levofloxacin). Empirical therapy was considered appropriate when the patient received at least one *in vitro* active antimicrobial agent within 24h following blood cultures before the susceptibility results were available, and the dosage and route of administration followed current medical standards. GNB were classified as MDR per prior consolidated definitions [12].

Overall mortality was defined as death by any cause within the first 30 days following the BSI onset. Death was considered related to BSI if it occurred either before symptoms or signs resolved, or within 7 days after the onset of bacteremia, with no other explanation.

### Microbiological methods

Blood samples were analyzed using the BACTEC 9240 system or Bactec FX system (Becton-Dickinson Microbiology Systems), with an incubation period of 5 days. Isolates were recognized by standard techniques. Antimicrobial susceptibility testing was performed by using a microdilution system (Microscan WalkAway Dade Behring, West Sacramento, CA or Phoenix system, Becton Dickinson, Franklin Lakes, NJ) or the Etest (AB Biodisk, Solna, Sweden/bioMérieux, Marcy l’Etoile, France). Current Clinical and Laboratory Standards Institute (CLSI) or EUCAST breakpoints for each year were used to define susceptibility or resistance to these antimicrobial agents; intermediate susceptibility was considered as resistance. ESBL were detected by MIC results and double-disk synergy test using disks containing cefotaxime, ceftazidime and cefepime that are applied to plates next to a disk with clavulanic acid.

#### Statistical analysis

Statistical analysis was performed using the SPSS v. 21.0 software tool (IBM SPSS, Chicago, IL, USA). Categorical variables were expressed numerically and as percentages, while continuous variables were presented as either means and standard deviations (SD) or medians and interquartile ranges (IQR), according to the distribution. Kolmogorov-Smirnov test was used for the normality analysis. Non-parametric approaches were undertaken given that most continuous variables were skewed. Differences between groups were evaluated using the Mann-Whitney U test for continuous variables and the χ^2^ test for categorical variables. Chi-square for trends was used to compare the different time spans. To define independent predictors of GNB CRBSIs, we performed multivariate regression analysis using binary logistic regression model–stepwise backward method. We constructed a regression model with the inclusion of the GNB CRBSIs as the dependent variable. For the independent variables, we chose those parameters that showed predictive value using univariate analysis. The calibration of the model was assessed by the Hosmer-Lemeshow goodness-of-fit test. The presence of collinearity was tested using the variance inflation factor (VIF) and tolerance. Interactions between variables were explored. In the case of multi-collinearity between parameters, we included in the model the parameter that showed greater predictive value using univariate analysis. Significance (p) was set at the value of 0.05.

## Results

### Incidence, patients characteristics, and descriptive epidemiology

A total of 27,083 BSI episodes were documented in the study period. Among them, 2,057 episodes were BSI in neutropenic oncological and hematological patients. Of those, BSI was catheter-related in 562 (27.3%) episodes, including 80 (14.2%) of which were polymicrobial. Further analysis was performed on the other 482 monomicrobial CRBSIs in neutropenic onco-hematological patients.

Proportion of CRBSIs among all BSIs decreased over time (p<0.001). Namely, in the first 5-year time span (1994–1998), 41.2% of all BSIs in this specific group of patients were CRBSIs. This percentage decreased during the second and last time spans to 21.2% and 15.8%, respectively. Patients’ characteristics are provided in Table 1. Median age, prior antibiotic treatment before BSI onset, and breakthrough BSI to beta-lactams increased over time. Shock at onset and overall mortality increased throughout the study period (p = 0.004 and p = 0.003, respectively).

**Table 1:** Demographic and clinical data in neutropenic onco-hematological patients with monomicrobial catheter-related bloodstream infection.

	Overall n = 482 (%)	1994–1998 n = 176 (%)	1999–2003 n = 72 (%)	2004–2008 n = 91 (%)	2009–2013 n = 92 (%)	2014–2018 n = 51 (%)	p-value
**Age–Median (IQR)**	49 (36–60)	44 (33–53)	45 (33–60)	55 (42–61)	53 (40–59)	55 (48–66)	**<0.001**
**Sex (M)**	294 (61.0)	101 (57.4)	51 (70.8)	56 (61.5)	62 (67.4)	24 (47.1)	0.858
**Underlying disease**							
** Acute leukemia**	226 (46.9)	87 (49.4)	40 (55.6)	44 (48.4)	25 (27.2)	30 (58.8)	0.628
** Other hematological diseases**	228 (47.3)	78 (44.3)	28 (38.9)	37 (40.7)	66 (71.7)	19 (37.2)	0.237
** Solid-organ tumor**	49 (10.2)	19 (10.8)	7 (9.7)	14 (15.4)	5 (5.4)	4 (7.8)	0.358
**HSCT**	174 (36.1)	96 (54.5)	22 (30.6)	17 (18.7)	25 (27.2)	14 (27.5)	**<0.001**
**Mc Cabe prognostic score**							
** Non-fatal**	114 (23.7)	46 (26.1)	13 (18.0)	16 (17.6)	24 (26.1)	15 (29.4)	0.325
** Ultimately fatal**	338 (70.1)	122 (69.2)	55 (76.4)	68 (74.7)	61 (66.3)	32 (62.7)	
** Rapidly fatal**	30 (6.2)	8 (4.5)	4 (5.6)	7 (7.7)	7 (7.6)	4 (7.8)	
**Type of catheter**							
** Peripheral**	55 (11.4)	25 (14.2)	3 (4.2)	7 (7.7)	9 (9.8)	11 (21.6)	0.511
** Central**	427 (88.6)	151 (85.8)	69 (95.8)	84 (92.3)	83 (90.2)	40 (78.4)	
**Femoral**	12 (2.5)	3 (1.7)	2 (2.8)	4 (4.4)	3 (3.3)	0 (0)	
**Subclavian**	27 (5.6)	13 (7.4)	3 (4.2)	7 (7.7)	3 (3.3)	1 (2.0)	
**Jugular**	346 (71.8)	131 (74.4)	60 (83.3)	60 (65.9)	63 (68.5)	32 (62.7)	
**Port-a-Cath**	42 (8.7)	4 (2.3)	4 (5.6)	13 (14.3)	14 (15.2)	7 (13.7)	
**Catheter culture**							
**Positive**	190 (39.4)	51 (29.0)	29 (40.3)	51 (56.0)	40 (43.5)	19 (37.3)	<0.001
**Negative**	73 (15.1)	21 (11.9)	7 (9.7)	7 (7.7)	14 (15.2)	24 (47.1)	
**Not performed**	219 (45.4)	104 (59.1)	36 (50.0)	33 (36.3)	38 (41.3)	8 (15.7)	
**Days after catheter placement (IQR)**	19 (12–44)	18 (11–40)	22 (14–22)	17 (10–30)	18 (12–57)	22 (15–77)	0.059
**Clinical presentation**							
**Fever**	460 (96.4)	174 (99.4)	69 (95.8)	87 (95.6)	84 (94.4)	46 (92)	**0.004**
**Shock**	21 (4.4)	2 (1.1)	2 (2.8)	7 (7.7)	6 (6.5)	4 (8.2)	**0.004**
**Prior antibiotic therapy**	282 (58.5)	84 (47.7)	55 (76.4)	44 (48.4)	64 (69.5)	35 (68.6)	**0.004**
**Beta-lactam**	145 (30.1)	31 (17.6)	26 (36.1)	19 (20.9)	45 (48.9)	24 (47.1)	**<0.001**
**Penicillins**^**±**^	48 (10.0)	7 (4.0)	12 (16.7)	7 (7.7)	16 (17.4)	6 (11.8)	**0.007**
**Third-generation cephalosporin**	48 (10.0)	14 (8.0)	6 (8.3)	6 (6.6)	14 (15.2)	8 (15.7)	**0.042**
**Carbapenem**	67 (13.9)	11 (6.3)	7 (9.7)	9 (9.9)	25 (27.2)	15 (29.4)	**<0.001**
**Aminoglycoside**	44 (9.1)	10 (5.7)	14 (19.4)	4 (4.4)	10 (10.9)	6 (11.8)	0.314
**Glycopeptide**	52 (10.8)	9 (5.1)	12 (16.7)	12 (13.2)	12 (13.0)	7 (13.7)	**0.031**
**Fluoroquinolone**	157 (32.6)	47 (26.7)	28 (38.9)	27 (29.7)	36 (39.1)	19 (37.3)	0.064
**>1 prior antibiotic**	123 (25.5)	25 (14.2)	18 (25)	17 (18.7)	40 (43.5)	23 (45.1)	**<0.001**
**Current antibiotic therapy**	353 (73.2)	134 (76.1)	59 (81.9)	52 (57.1)	71 (77.2)	37 (72.5)	**0.003**
**Beta-lactam**	165 (34.2)	54 (30.7)	23 (31.9)	25 (27.5)	38 (41.3)	25 (49.0)	**0.015**
**Penicillins**^**±**^	30 (6.2)	13 (7.4)	6 (8.3)	4 (4.4)	5 (5.4)	2 (3.9)	0.247
**Third-generation cephalosporin**	51 (10.6)	19 (10.8)	6 (8.3)	5 (5.5)	13 (14.3)	8 (15.7)	0.328
**Carbapenem**	82 (17.0)	21 (11.9)	10 (13.9)	16 (17.6)	20 (21.7)	15 (29.4)	**0.001**
**Aminoglycoside**	40 (8.3)	24 (13.6)	7 (9.7)	2 (2.2)	5 (5.4)	2 (3.9)	**0.002**
**Glycopeptide**	73 (15.1)	23 (13.1)	11 (15.3)	19 (20.9)	12 (13.0)	8 (15.7)	0.600
**Fluoroquinolone**	215 (44.6)	86 (48.9)	38 (52.8)	28 (30.8)	44 (47.8)	19 (37.3)	0.100
**Combination therapy**	131 (27.2)	49 (27.8)	19 (26.4)	27 (29.0)	27 (29.3)	17 (33.3)	0.680
**IEAT**	145 (30.1)	63 (36)	31 (43.1)	22 (24.2)	15 (16.1)	14 (27.5)	**0.001**
**Overall mortality**	37 (7.8)	8 (4.5)	2 (2.8)	8 (8.9)	13 (14.6)	6 (11.8)	**0.003**

**M:** Male; **IQR**: Interquartile range; **HSCT:** Hematopoietic stem-cell transplant; **COPD:** Chronic obstructive pulmonary disease; **BSI:** Bloodstream infection; **IEAT:** Inappropriate empirical antibiotic treatment.

^±^Consisting of Piperacillin-Tazobactam, Amoxicillin, Ampicillin, Amoxicillin-Clavulanate and Cloxacillin.

**Fig 1:**
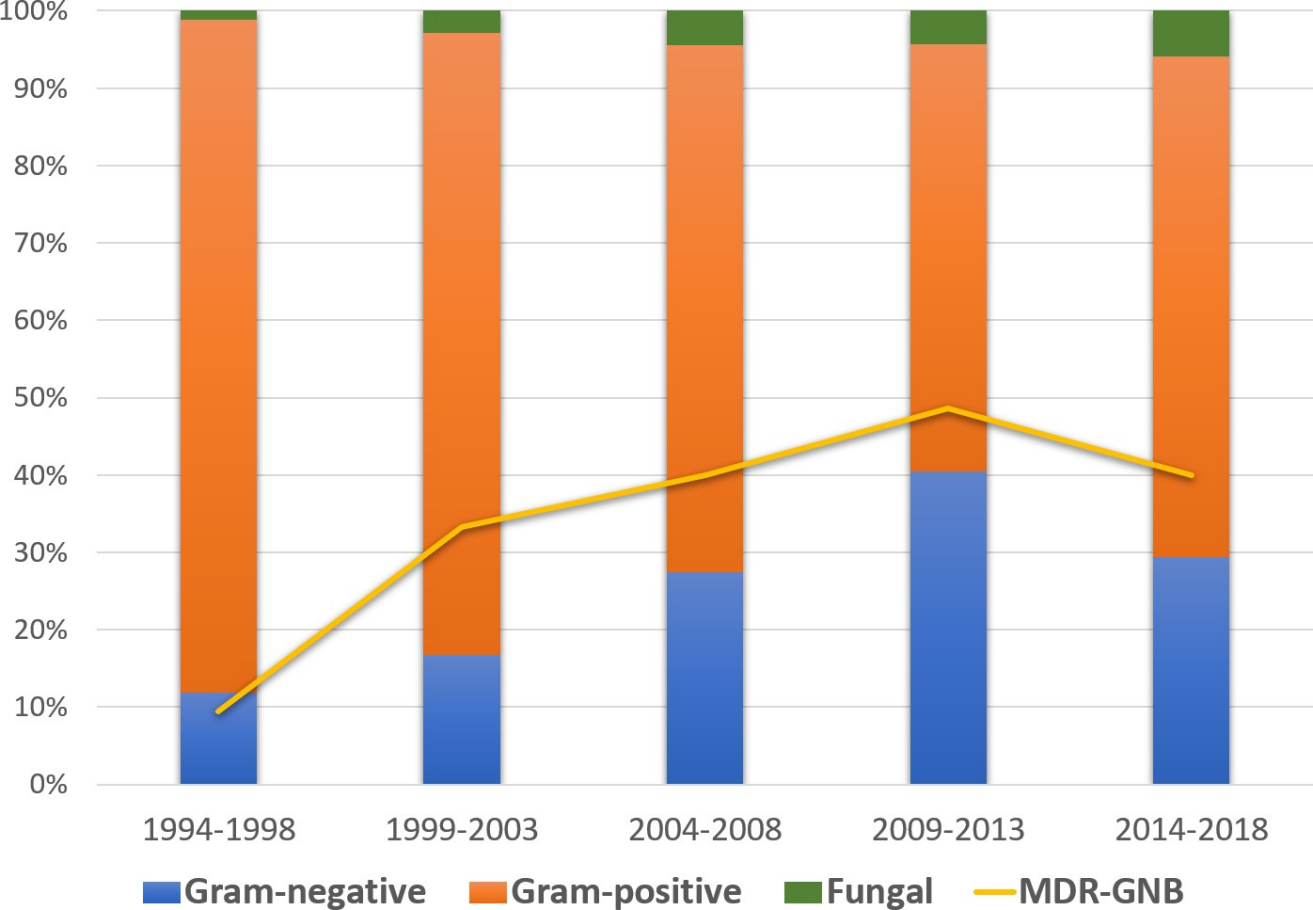
Evolution of Gram-positive cocci, Gram-negative bacilli, candidemia, and rates of MDR among GNB, throughout the study period.

Causative agents of CRBSI throughout the study period are shown in Table 2. Overall, Gram-positive (GP) bacteremia was detected in 357 (74.1%), GN in 110 (22.8%), and candidemia in 15 (3.1%), of which 10 were non-albicans fungemias. A significant decrease in rates of Gram-positive cocci (GPC) and increase in rates of GNB and candidemia were documented (p<0.001, p<0.001 and p = 0.039, respectively). Among GP, the proportion of coagulase-negative staphylococci (CoNS) decreased over time from 94.1% in the first time span to 78.8% in the last, while the proportion of *Enterococcus* spp. increased from 2.0% to 18.2% (p<0.001), particularly due to *E*. *faecium*. GNB-CRBSIs were most frequently caused by *P*. *aeruginosa* and *E*. *coli* in all observed time-spans. An increase in the percentage of *P*. *aeruginosa* in the last three time-spans was observed, comprising up to 40% of all GNB isolates. Additionally, there was a significant increase among MDR-GNB CRBSI episodes in the last three time-spans, mainly caused by an increase in MDR-PA isolates and ESBL-producers *E*. *coli* and *K*. *pneumoniae*. Fig 1 shows the evolution of GPC, GNB, and candidemia throughout the study period, as well as the rates of MDR among GNB

**Table 2:** Epidemiology of CRBSI in oncohematological patients during the evaluated period.

	Overall n = 482 (%)	1994–1998 n = 176 (%)	1999–2003 n = 72 (%)	2004–2008 n = 91 (%)	2009–2013 n = 92 (%)	2014–2018 n = 51 (%)	p-value
**Gram-positive (GPC)**	357 (74.1)	153 (86.9)	58 (80.5)	62 (68.1)	51 (54.8)	33 (64.7)	**<0.001**
***S*. *aureus***	17 (4.8*)	5 (3.3*)	3 (5.2*)	5 (8.1*)	3 (5.9*)	1 (3.0*)	0.499
**MRSA**	4 (23.5^†^)	2 (40.0^†^)	0 (0.0^†^)	1 (20.0^†^)	1 (33.0^†^)	0 (0.0^†^)	-
**CoNS**	322 (90.2*)	144 (94.1*)	55 (94.8*)	52 (83.9*)	45 (88.2*)	26 (78.8*)	**0.012**
***Enterococcus* spp.**	16 (4.5*)	3 (2.0*)	0 (0.0*)	4 (6.5*)	3 (5.9*)	6 (18.2*)	**<0.001**
***E*.*faecalis***	8 (50^†^)	2 (66.6^†^)	0 (0.0^†^)	3 (75.0^†^)	2 (66.6^†^)	1 (16.7^†^)	-
***E*.*faecium***	8 (50^†^)	1 (33.3^†^)	0 (0.0^†^)	1 (25.0^†^)	1 (33.3^†^)	5 (83.3^†^)	-
***Streptococcus* spp.**	2 (0.6*)	1 (0.7)	0 (0.0*)	1 (1.6)	0 (0.0*)	0 (0.0*)	-
**Gram-negative (GNB)**	110 (22.8)	21 (11.9)	12 (16.7)	25 (27.5)	37 (40.2)	15 (29.4)	**<0.001**
***E*. *coli***	29 (26.4**)	6 (28.6**)	3 (25.0**)	7 (28.0**)	8 (21.6**)	5 (33.3**)	0.944
**ESBL**	8 (27.6^†^)	0 (0.0^†^)	0 (0.0^†^)	3 (42.9^†^)	4 (50.0^†^)	1 (20.0^†^)	0.124
***P*. *aeruginosa***	36 (32.7**)	5 (23.8**)	2 (16.7**)	8 (32.0**)	15 (40.5**)	6 (40.0**)	0.099
**MDR (including XDR isolates)**	13 (36.1^†^)	0 (0.0^†^)	0 (0.0^†^)	5 (62.5^†^)	6 (40.0^†^)	2 (33.3^†^)	0.282
**XDR**	12 (33.3^†^)	0 (0.0^†^)	0 (0.0^†^)	4 (50.0^†^)	6 (40.0^†^)	2 (33.3^†^)	0.215
***K*. *pneumoniae***	9 (8.2**)	1 (4.8**)	0 (0.0**)	3 (12.0**)	4 (10.8**)	1 (6.7**)	0.441
**ESBL**	3 (33.3^†^)	0 (0.0^†^)	0 (0.0^†^)	0 (0.0^†^)	3 (75.0^†^)	0 (0.0^†^)	-
***S*. *maltophilia***	14 (12.7**)	2 (9.5**)	4 (33.3**)	2 (8.0**)	5 (13.5**)	1 (6.7**)	0.567
***Acinetobacter* spp.**	4 (3.6**)	2 (9.5**)	1 (8.3**)	0 (0.0**)	1 (2.7**)	0 (0.0**)	0.086
***Enterobacter* spp.**	3 (2.7**)	0 (0.0**)	0 (0.0**)	1 (4.0**)	0 (0.0**)	2 (13.3**)	-
**MDR-GNB**	40 (36.3)	2 (9.5)	4 (33.3)	10 (40.0)	18 (48.6)	6 (40.0)	**0.039**
***Candida* spp.**	15 (3.1)	2 (1.1)	2 (2.8)	4 (4.4)	4 (4.3)	3 (5.9)	**0.039**
**Non*-albicans Candida***	10 (66.7)	1 (50.0)	2 (100.0)	3 (75.0)	2 (50.0)	2 (66.7)	0.182

^*^Percentage within the GPC

**Percentage within GNB

^**†**^Percentage among their species, CoNS–coagulase-negative staphylococci, ESBL–Extended-spectrum beta-lactamase, MRSA–methicillin-resistant *Staphylococcus aureus*, MDR–multidrug-resistant, XDR–extensively drug-resistan.

### Risk factors for GNB CRBSIs

Table 3 details differences between patients with GPC and GNB CRBSIs. Patients with GNB CRBSIs were significantly older, had greater length of in situ catheter, and presented with septic shock more frequently (p = 0.006, p = 0.013 and p = 0.002, respectively). Stem cell transplant recipients had more frequently GPC than GNB (p = 0.007), while GNB were significantly more frequent in patients with solid neoplasms (p = 0.050). Previous treatment with beta-lactams (p = 0.006) and current treatment with glycopeptides (p = 0.034) were related to GNB, while current treatment with fluoroquinolones was associated with GPC (p = 0.004).

**Table 3:** Differences between CRBSIs caused by Gram-positive cocci and Gram-negative bacilli.

	GPC n = 357 (%)	GNB n = 110 (%)	p-value
**Age–Median (IQR)**	48 (35–59)	54 (42–61)	**0.006**
**Male**	221 (61.9)	64 (57.7)	0.437
**Hematological malignancy**	339 (95.0)	102 (91.9)	0.245
**Solid neoplasm**	31 (8.7)	17 (15.3)	**0.050**
**HSCT**	141 (39.5)	28 (25.2)	**0.007**
**Catheter culture**			
**Positive**	138 (38.7)	43 (38.7)	0.219
**Negative**	47 (13.2)	23 (20.7)	
**Not performed**	172 (48.2)	45 (40.9)	
**Type of catheter**			
**Femoral**	10 (2.8)	1 (0.9)	**<0.001**
**Subclavian**	17 (4.9)	**9 (8.4)**	
**Jugular**	**266 (76.9)**	60 (56.1)	
**Port-a-cath**	17 (4.9)	**23 (21.5)**	
**PVC**	36 (10.4)	14 (13.1)	
**Days after catheter placement–Median (IQR)**	18 (11–40)	24 (13–60)	**0.013**
**Clinical presentation**			
**Fever**	342 (96.6)	105 (96.3)	0.889
**Shock**	9 (2.5)	11 (10.0)	**0.002**
**Prior corticosteroid therapy**	124 (35.8)	43 (41.7)	0.276
**Prior antibiotic therapy**			
**Beta-lactam**	93 (26.1)	44 (39.6)	**0.006**
**Penicilins**^**±**^	26 (7.0)	22 (20.0)	**<0.001**
**Aminoglycoside**	27 (7.6)	15 (13.5)	**0.055**
**Glycopeptide**	37 (10.4)	14 (12.6)	0.507
**Fluoroquinolone**	118 (33.1)	34 (30.6)	0.634
**Current antibiotic therapy**			
**Beta-lactam**	116 (32.5)	37 (33.3)	0.908
**Aminoglycoside**	30 (8.4)	10 (9.0)	0.842
**Glycopeptide**	45 (12.6)	23 (20.7)	**0.034**
**Fluoroquinolone**	175 (49.0)	37 (33.3)	**0.004**
**IEAT**	106 (29.7)	22 (20)	**0.012**
**Overall mortality**	19 (5.4)	16 (14.4)	**0.002**
**Related mortality**	13 (3.7)	8 (7.2)	0.124

**GPC:** Gram-positive cocci; **GNB:** Gram-negative bacilli; **IQR**: Interquartile range; **HSCT:** Hematopoietic stem-cell transplant; **PVC:** Peripheral venous catheter; **IEAT:** Inappropriate empirical antibiotic treatment.

^±^Consisting of piperacillin-tazobactam, amoxicillin, ampicillin, amoxicillin-clavulanate and cloxacillin

**Fig 2:**
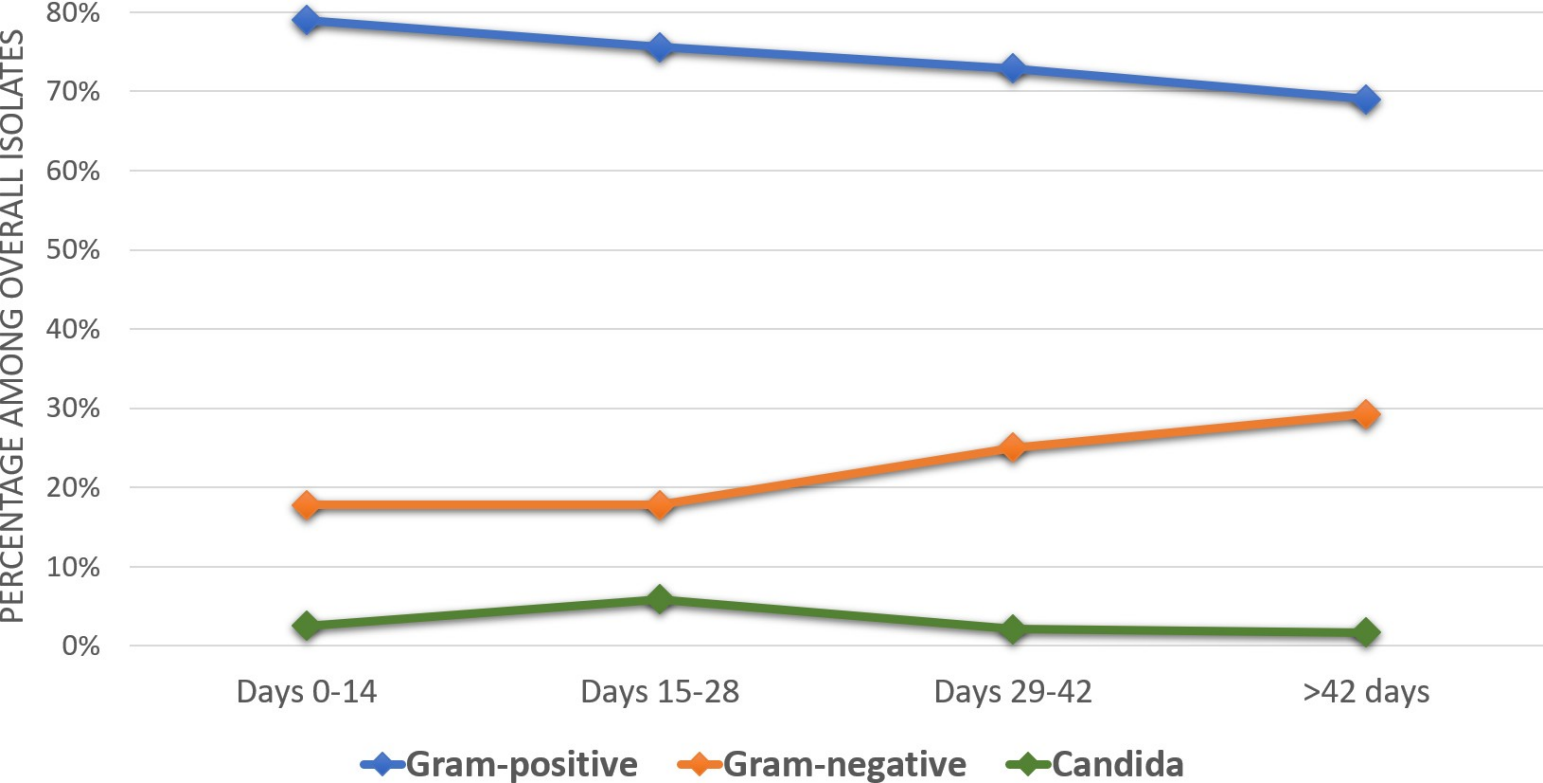
Epidemiological changes related to the duration of in situ catheter.

Independent factors associated with GNB-CRBSIs were: longer duration of in situ catheter (OR 1.007; 95% CI 1.004–1.011), older age (OR 1.016; 95% CI 1.001–1.033), previous antibiotic treatment with penicillins (consisting of piperacillin-tazobactam, amoxicillin, ampicillin, amoxicillin-clavulanate and cloxacillin) (OR 2.716; 95% CI 1.306–5.403) and current antibiotic treatment with glycopeptides (OR 1.931; 95% CI 1.001–3.306) (Table 4). Fig 2 displays the epidemiological changes related to the duration of in situ catheter.

**Table 4:** Independent risk factors for GNB in CRBSI in neutropenic oncological and hematological patients.

	Adjusted odds ratio (95% CI)	p-value
**Older age**	1.016 (1.001–1.033)	**0.043**
**Longer duration of in-situ catheter**	1.007 (1.004–1.011)	**<0.001**
**Prior antibiotic therapy with penicilins**^**±**^	2.716 (1.366–5.403)	**0.004**
**Current antibiotic therapy with glycopeptides**	1.931 (1.001–3.306)	**0.041**

^±^Consisting of piperacillin-tazobactam, amoxicillin, ampicillin, amoxicillin-clavulanate and cloxacillin.

### IEAT and mortality

Approximately as many as one-third of patients (30.7%) received inappropriate empirical treatment. Table 5 details rates of IEAT and mortality according to different bacterial isolates. The highest percentage of inappropriate empirical treatment was detected for MDR *P*. *aeruginosa* (76.9%) and *Stenotrophomonas maltophilia* (92.9%), followed by CoNS (32.9%) and *Enterococcus* spp. (31.3%).

**Table 5:** Most frequent microbiological isolates associated with inappropriate antibiotic therapy and high mortality.

Microorganism	n	IEAT n (%)	Mortality in IEAT n (%)	Mortality in AEAT n (%)	Overall mortality n (%)	p-value
**Gram-positive (GPC)**	357	106 (30.1)	12 (5)	7 (6.2)	19 (5.4)	0.649
***S*. *aureus***	17	2 (11.8)	1 (50.0)	2 (13.3)	3 (17.6)	0.331
**MRSA**	4	2 (50.0)	1 (50)	0	1 (25.0)	1.000
**CoNS**	310	101 (32.9)	6 (5.9)	8 (3.9)	14 (4.5)	0.433
***Enterococcus* spp.**	16	4 (25.0)	0	2 (16.7)	2 (12.5)	1.000
***E*.*faecium***	8	2 (25.0)	0	2 (33.3)	2 (25.0)	1.000
**Gram-negative (GNB)**	110	22 (20.0)	6 (27.3)	9 (10.2)	15 (13.6)	**0.037**
***E*. *coli***	29	1 (3.4)	0	4 (14.3)	4 (13.8)	1.000
**ESBL**	8	1 (12.5)	0	3 (42.9)	3 (37.5)	1.000
***P*. *aeruginosa***	36	9 (25.0)	3 (33.3)	2 (7.4)	5 (13.9)	0.088
**MDR**	13	9 (69.2)	3 (33.3)	1 (25)	4 (30.8)	1.000
***K*. *pneumoniae***	9	0	0	1 (11.1)	1 (11.1)	-
**ESBL**	3	0	0	0	0	-
***S*. *maltophilia***	14	11 (78.6)	3 (27.3)	1 (33.3)	4 (28.6)	1.000

**IEAT:** Inappropriate empirical antibiotic treatment; **AEAT:** Appropriate empirical antibiotic treatment; **MRSA:** Methicillin-resistant *Staphylococcus aureus;* **CoNS**: Coagulase-negative Staphylococci; **ESBL**: Extended-spectrum beta-lactamase; **MDR**: Multidrug-resistant; **XDR:** Extremely drug-resistant.

Overall mortality rate was 7.8%, with a significantly greater mortality rate among GNB than GPC CRBSI (14.4% vs 5.4%, p = 0.002). Mortality rate was significantly higher in the last three time spans, in parallel with the growing absolute number of infections caused by MDR-GNB. Higher mortality rate among GNB than GPC is predominantly due to increased mortality rates among patients with ESBL-producing *E*. *coli* (37.5%), MDR *P*. *aeruginosa* (30.8%) and *S*. *maltophilia* infection (28.6%). In GPC, the highest mortality rate was detected for *E*. *faecium* and MRSA infection.

## Discussion

The most important findings of our study are the following: 1) CRBSIs proportion among all BSI decreased over time; 2) CRBSIs epidemiology is changing with increasing rates of GNB, 3) the absolute number of MDR-GNB infections increased over time; 4) independent risk factors for GNB CRBSI were: older age, longer duration of in situ catheter, previous antibiotic treatment with penicillins and current antibiotic treatment with glycopeptides; 5) approximately one third of patients received inappropriate empirical antibiotic treatment; and 6) CRBSI mortality rate was high and increased significantly over the study period, in parallel with the increase in MDR-GNB infections.

Our study showed a decreasing trend in the incidence of CRBSI in neutropenic onco-hematological patients. This could be the result of both a development in asepsis and antisepsis preventive measures during catheter placement and management, and health professionals’ education. This decreasing incidence has also been noted in other studies in general population patients [13–15], although to the best of our knowledge, this is one of the firsts studies showing these results in onco-hematological patients. Chaftari et al. compared CRBSI in two different periods separated by over a decade [16]. Like our results, these authors showed a significant decrease in the rates of CRBSI between both periods, as well as an increase in the rates of GNB and proportional decrease in GPC. In this study, GNB-CRBSI also ocurred after a much longer duration of catheter placement.

However, as CRBSI continue to cause significant morbidity in oncohematological patients, lock therapies or coated catheters may be considered in the future to prevent these biofilm-mediated infections [17]. Also, implementation and maintenance of bundles, is a cost-effective strategy to prevent CRBSI [7].

Although the vast majority of CRBSI occur in patients with central venous catheters, the impact of peripherally inserted catheters is probably underestimated [18]. In this sense, in this study, peripheral venous catheters caused 11.4% of all cases. This is important, as the prevalence of Gram-negative CRBSI seems to be increasing in these cases [14].

In the last years, a shift towards a progressive increase in GNB in the overall BSI episodes in oncohematological patients has been described [12,19–21], with some studies reporting even a predominance of GNB [22–24]. Additionally, some studies have identified neutropenia to be an independent risk factor for presenting a CRBSI caused by GNB [25]. The specifical changes reported in this study, with a progressive linear increase in the percentage of GNB among CRBSIs in neutropenic patients, as well as in other CRBSI studies in the general population [13,26,27], might be at least partially explained by the increasing age and complexity of patients.

It has been hypothesized that this shift to GNB could also be explained by the discontinuation of quinolone prophylaxis; following the rising resistance among GNB [1]. However, in our center, quinolone prophylaxis is still routinely given to all patients with expected neutropenia longer than 10 days, and yet, an increase in GNB proportion has been documented in CRBSI and overall episodes [12].

The most common isolates among GNB were *P*. *aeruginosa* and *E*. *coli*, with an increase in the percentage of *P*. *aeruginosa* in the last three time spans, comprising up to 40% of all GNB isolates. Most importantly, a significant increase occurred in both absolute numbers and percentages of MDR and XDR isolates of *P*. *aeruginosa* and ESBL-producing *E*. *coli* and *K*. *pneumoniae* in the last three time spans. This worrisome trend of increasing resistance among GNB isolates has been previously reported [23,28], and is especially pronounced for *P*. *aeruginosa*, comprising 36.1% of MDR isolates. Similar percentages of MDR *P*. *aeruginosa* have already been described in other studies involving oncohematological patients [23,29,30]. Our study highlights the fact that GNB, including MDR isolates, are an increasing cause of infections, even when the catheter is the suspected source. All these facts should be taken into account when updating the international guidelines or specific protocols for managing both CRBSIs and neutropenic fever.

We identified risk factors for GNB CRBSI. Some of these factors, such as longer catheter duration or prior antibiotic therapy had already been identified in previous studies [14,15]. Some other studies have also identified the insertion site to be related with the risk of some specific GNB [31]. Considering these risks factors is mandatory to optimize empirical antibiotic treatment when catheter-related infection is suspected. In our study, inappropriate empirical treatment was frequent, and overall mortality increased, partly due to the irruption of GNB-MDR isolates. Catheter-removal seems mandatory in CRBSI caused by GNB, as it has been associated with significantly increased microbiological cure and decreased mortality [32]. Unfortunately, we did not have data on catheter removal in our cohort, and were thus unable to evaluate the impact of such measure in the prognosis.

The strengths of our study are the large number of patients included, prospective data collection, and the extensive clinical and microbiological data gathered by an experienced team. However, the major limitation of the study is fact that it was conducted at a single center; bearing in mind that microbiological epidemiology varies significantly in different geographical contexts.

In conclusion, we found a decreased proportion of CRBSIs among all BSIs, as well as a changing CRBSIs epidemiology. The most important change found was the rising rate of GNB over time as well as the increased in MDR-GNB episodes. Percentages of IEAT and mortality were high and rose in parallel with the increase of MDR-GNB infections. Longer duration of in situ catheter, older age, prior antibiotic treatment with penicillins, and current antibiotic treatment with glycopeptides, were independent predictors of GNB-CRBSI. Improving empirical antibiotic treatment, especially in patients at risk of MDR-GNB infections is mandatory.

## Supporting information

S1 Database (SAV)
